# Leveraging family history in genetic association analyses of binary traits

**DOI:** 10.1186/s12864-022-08897-8

**Published:** 2022-10-01

**Authors:** Yixin Zhang, James B. Meigs, Ching-Ti Liu, Josée Dupuis, Chloé Sarnowski

**Affiliations:** 1 Department of Biostatistics, Boston University School of Public Health, Boston, MA USA; 2 Division of General Internal Medicine, Massachusetts General Hospital, Boston, MA USA; 3 Department of Medicine, Harvard Medical School, Boston, MA USA; 4 Department of Epidemiology, Biostatistics and Occupational Health, McGill University, Montréal, Québec Canada; 5 Department of Epidemiology, Human Genetics, and Environmental Sciences, The University of Texas Health Science Center, School of Public Health, Houston, TX USA

**Keywords:** Family history, GWAS, Meta-analysis

## Abstract

**Background:**

Considering relatives’ health history in logistic regression for case–control genome-wide association studies (CC-GWAS) may provide new information that increases accuracy and power to detect disease associated genetic variants. We conducted simulations and analyzed type 2 diabetes (T2D) data from the Framingham Heart Study (FHS) to compare two methods, liability threshold model conditional on both case–control status and family history (LT-FH) and Fam-meta, which incorporate family history into CC-GWAS.

**Results:**

In our simulation scenario of trait with modest T2D heritability (h^2^ = 0.28), variant minor allele frequency ranging from 1% to 50%, and 1% of phenotype variance explained by the genetic variants, Fam-meta had the highest overall power, while both methods incorporating family history were more powerful than CC-GWAS. All three methods had controlled type I error rates, while LT-FH was the most conservative with a lower-than-expected error rate. In addition, we observed a substantial increase in power of the two familial history methods compared to CC-GWAS when the prevalence of the phenotype increased with age. Furthermore, we showed that, when only the phenotypes of more distant relatives were available, Fam-meta still remained more powerful than CC-GWAS, confirming that leveraging disease history of both close and distant relatives can increase power of association analyses. Using FHS data, we confirmed the well-known association of *TCF7L2* region with T2D at the genome-wide threshold of *P*-value < 5 × 10^–8^, and both familial history methods increased the significance of the region compared to CC-GWAS. We identified two loci at 5q35 (*ADAMTS2*) and 5q23 (*PRR16*), not previously reported for T2D using CC-GWAS and Fam-meta; both genes play a role in cardiovascular diseases. Additionally, CC-GWAS detected one more significant locus at 13q31 (*GPC6*) reported associated with T2D-related traits.

**Conclusions:**

Overall, LT-FH and Fam-meta had higher power than CC-GWAS in simulations, especially using phenotypes that were more prevalent in older age groups, and both methods detected known genetic variants with lower *P*-values in real data application, highlighting the benefits of including family history in genetic association studies.

**Supplementary Information:**

The online version contains supplementary material available at 10.1186/s12864-022-08897-8.

## Background

Traditional genome-wide association studies assessing the association with binary traits using a logistic regression (CC-GWAS) are based on each study individual’s genotypes and case–control status. Phenotypic status of relatives who are not genotyped is also expected to be associated with participants’ genotypes based on the rules of genetic inheritance. While readily available but often ignored, family history provides additional valuable information that may increase the accuracy and power of association tests. To leverage family history in genetic association studies, different approaches have been proposed. A popular technique focuses on imputing missing genotypes for individuals based on their relatives’ genotypes and a known pedigree structure [1–4]. According to these imputation methods, incorporating predicted genotypes of relatives increases the power of genetic association studies. One specific study used the Framingham Heart Study (FHS) cohort to demonstrate that using identity-by-descent information in families to impute genotypes strengthened the association signals for known disease loci [2]. However, there are several major disadvantages to the imputation of non-genotyped individuals, as imputation can be very computationally expensive depending on the number of SNPs, and the accuracy of the estimated genotypes varies among different types of studies.

In this paper, we examined two proposed approaches to directly integrate the phenotypes of ungenotyped relatives into association analysis, without any additional time-consuming imputations or further costly genotyping. The “liability threshold model conditional on both case–control status and family history” (LT-FH) method [5] replaces binary case–control status with posterior mean genetic liability scores based on each individual’s and first-degree relatives’ phenotypes. Another method [6] proposes a meta-analysis framework (referred to as Fam-meta in this paper) to combine test statistics from two independent regression analyses, one involving genotyped individuals, the other involving their relatives with phenotypic information only. It has been shown empirically [5, 6] that each familial history approach substantially increases power to detect risk loci associated with diseases compared to CC-GWAS, though the two approaches have not yet been compared directly. In this study, we assessed and contrasted the performance of LT-FH and Fam-meta by first conducting simulations, and then association analysis of type 2 diabetes (T2D) in the FHS dataset.

## Methods

### Method overview

#### LT-FH

Details regarding the LT-FH method can be found elsewhere [5]. In LT-FH, the first step is to compute a posterior mean genetic liability for each individual, based on the individual’s disease status as well as any available parent or sibling disease status. The genetic liabilities are assumed to follow a multivariate normal distribution:

$$\left(\begin{array}{c}{\epsilon }_{o,e}\\ {\epsilon }_{o,g}\\ {\epsilon }_{p1}\\ {\epsilon }_{p2}\\ {\epsilon }_{s}\end{array}\right)\sim {N}_{5}\left(\left(\begin{array}{c}0\\ 0\\ 0\\ 0\\ 0\end{array}\right),\left(\begin{array}{ccccc}1-{h}^{2}& 0& 0& 0& 0\\ 0& {h}^{2}& {0.5h}^{2}& 0.5{h}^{2}& {0.5h}^{2}\\ 0& {0.5h}^{2}& 1& 0& {0.5h}^{2}\\ 0& {0.5h}^{2}& 0& 1& {0.5h}^{2}\\ 0& {0.5h}^{2}& {0.5h}^{2}& {0.5h}^{2}& 1\end{array}\right)\right)$$

Which includes the environmental (${\epsilon }_{o,e}$) and genetic (${\epsilon }_{o,g}$) components of the liabilities of offspring, the liabilities of parent (${\epsilon }_{p1},{\epsilon }_{p2}$), and the liabilities of siblings (${\epsilon }_{s}$). Given the disease status of an individual ${Z}_{0}$, his parents ${Z}_{p1},{Z}_{p2}$, and siblings ${Z}_{s}$, The posterior mean genetic liabilities $E[{\epsilon }_{o,g}|{Z}_{0},{Z}_{p1},{Z}_{p2},{Z}_{s}]$ for every possible configuration of case–control status and family history are estimated with Monte Carlo integration. In the second step, an association test is conducted between the continuous liabilities and genotypes. The association statistics can be obtained using multiple models, such as linear regression, score test, or linear mixed effect models.

#### Fam-meta

Fam-meta considers two independent association tests that have been previously described elsewhere [6]. The first analysis involves probands, or participants who have both genotypes and phenotype information available. A logistic regression can be fitted based on standard likelihood for case–control. The second analysis involves relatives who only have phenotypic information. This logistic regression is based on the likelihood of observing a relative’s case–control status, conditioning on both the proband’s case–control status as well as the probands’ phenotypes. The regression coefficients and variances from both regressions are then combined in a meta-analysis framework with optimal (inverse variance) weights:

$${T}_{meta}=\frac{\frac{1}{\widehat{var}(\widehat{{\beta }^{P}})}\widehat{{\beta }^{P}}+\frac{2\phi }{\widehat{var}(\widehat{{\beta }^{R}})}\widehat{{\beta }^{R}}}{\sqrt{\frac{1}{\widehat{var}(\widehat{{\beta }^{P}})}+\frac{4{\phi }^{2}}{ \widehat{var}(\widehat{{\beta }^{R}})}}}$$

where $\widehat{{\beta }^{P}}$ is the beta coefficient from the probands’ regression, $\widehat{{\beta }^{R}}$ is the beta coefficient from the relatives’ regression, and $\phi$ is the kinship coefficient between the participant and the relative.

### Simulations

#### Main models

We conducted simulations to directly compare the performance of LT-FH and Fam-meta, while CC-GWAS results served as a reference. Three main models were utilized to generate phenotypes: a null model without SNP effects (model 1), a model with variants contributing to disease liability (model 2), and a model with an additional age by genotype interaction (model 3). To evaluate each model, we first generated 400 nuclear families consisting of parents and offspring for each iteration, where 200 families have two children, and 200 families have three children (*N* = 1800). Sex was randomly assigned for the children, and age was assigned using the following rules: each child’s age was generated under a continuous uniform distribution between 18 to 45 years old; the mother was 20–45 years older than the oldest child; the father was within five years of the mother’s age, and had to be at least 20 years older than the oldest child.

For the null model (model 1), we simulated one continuous disease trait using the following formula:

$$Y=0.015age+0.45sex+\gamma +\varepsilon$$

Age and sex explained 10% and 5% of the total phenotypic variance, respectively. The polygenic component $\gamma$ followed a multivariate normal distribution with mean 0 and covariance ${\phi \sigma }_{G}^{2}$, where $\phi$ was the kinship matrix of one family, and ${\sigma }_{G}^{2}$ was set to 0.2. The random error $\varepsilon$ was normally distributed with variance ${\sigma }_{E}^{2}$=0.65.

In the second model (model 2), we incorporated eight independent causal variants with the following minor allele frequencies (MAFs): 1%, 2%, 5%, 10%, 20%, 30%, 40%, 50% (See Additional File 1 for description and results of additional scenarios where less frequent variations, or variants in linkage disequilibrium (LD) generated using HAPGEN2 [7] and 1000 Genomes reference panel were used). We first assigned parents’ genotypes under Hardy–Weinberg equilibrium, i.e., the probability of having 0, 1, or 2 minor alleles are p^2^, 2pq, q^2^, respectively, where p is the MAF. Then, we determined the children’s genotypes through gene dropping, assuming that each parent passes down one of their alleles to their offspring with an equal chance of selecting either allele. Phenotypes were generated as follows:

$$Y={\sum }_{k=1}^{8}{\beta }_{k}\left({g}_{k}\right)+0.015age+0.45sex+\gamma +\varepsilon$$

We assumed trait heritability of 0.28, including 1% of total phenotypic variance explained by each of the eight causal variants, and variability due to the polygenic component $\gamma$ described in model 1. The ${\beta }_{k}$ was calculated using $\sqrt{\frac{1\%}{2\times MAF\times (1-MAF)}}$. Ten percent of total variance was explained by age, and 5% of total variance was explained by sex. The remaining variance was explained by the normally distributed random error $\varepsilon$, where ${\sigma }_{E}^{2}$=0.57.

We incorporated into the third model (model 3) an interaction between age and genotype of causal variants in addition to the eight causal variants:

$$Y= 0.02Age({\sum }_{k=1}^{8}{\beta }_{k}\left({g}_{k}\right))+{\sum }_{k=1}^{8}{\beta }_{k}\left({g}_{k}\right)+0.015Age+0.45Sex+\gamma +\varepsilon$$

Causal variants were generated the same way as in model 2. We reduced the proportion of phenotypic variance explained by each causal variant from 1% to 0.5% so that the genotype by age interaction term and the genotype term each explained 4% of the total phenotypic variance (see Additional File 1 for details regarding an additional scenario where we simulated phenotype with a larger age effect).

The continuous trait Y was transformed to a binary case–control status for all three models using a threshold model with a disease prevalence of 0.3. To evaluate type I error rate of each model, we generated eight independent, non-causal variants with the same allele frequencies as the causal variants, and assessed their individual association with case–control status using CC-GWAS, LT-FH, and Fam-meta. For model 1, we completed 50,000 simulation replicates under H_0_: there is no association between non-causal variants and case–control status, then calculated the proportion of replicates with *P*-value less than 5%, 1%, and 0.5%; for models 2 and 3, we completed 5000 replicates and used a 5% alpha level. To determine power for models 2 and 3, we examined the association between the binary trait of each model and the causal SNPs using CC-GWAS, LT-FH, and Fam-meta. We ran 5000 simulation replicates under H_1_: there is an association between causal variants and case–control status, and calculated power as the proportion of replicates with *P*-value less than 5%. Note that in all association tests, we only used offspring (*N* = 1000) for CC-GWAS, as if the parents’ genotypes were unavailable but their phenotypes (case–control statuses) were. LT-FH and Fam-meta each leveraged the phenotypes of parents accordingly (see descriptions of each method above).

#### Use of more distant relatives

To test the influence of leveraging more distant relatives’ information compared to first-degree relatives, we used the phenotypes of grandparents instead of parents as the available family history information. We only tested this scenario with CC-GWAS and Fam-meta, because LT-FH is not designed to incorporate second-degree relatives. A new family structure was utilized, where each family still comprised two parents with two or three offspring, but each parent also had their respective parents, totaling four grandparents. Similar to previous simulations, there were 200 families with two grandchildren and 200 families with three grandchildren (*N* = 3400). Using the new pedigree, we simulated phenotypes under model 2, and ran association tests using the offspring (*N* = 1000) for CC-GWAS. In terms of the relatives regression conducted in Fam-meta, we considered two sample sizes: first, all grandparents (*N* = 1600), and second, only one pair of randomly selected grandparents (*N* = 800). The purpose of this scenario was to compare whether including a larger number of grandparents in association analyses would increase power. For each sample size, we ran 5000 iterations.

### Application of both methods to the analysis of T2D in FHS

#### Description of the FHS

The FHS is an ongoing longitudinal cohort study that began in 1948, with an initial enrollment of 5209 first-generation participants who were mainly of European descent. Over the years, the cohort has grown substantially to include offspring (Offspring cohort) and grandchildren (Gen 3 cohort), while numerous health conditions have been monitored to assess cardiovascular diseases and their risk factors. Both LT-FH and Fam-meta were applied to the same three generations of FHS participants, with T2D case–control status available for most participants. T2D was defined as having at least one of the following conditions: fasting glucose ≥ 7 mmol/L after ≥ 8 h, Hemoglobin A1c ≥ 6.5%-units, 2-hour glucose by an oral glucose tolerance test ≥ 11.1 mmol/L, non-fasting glucose ≥ 11.1 mmol/L, physician-diagnosed diabetes, or use of antidiabetic medication. Participants with known type 1 diabetes were excluded. In the case where a second-generation participant’s parent was not part of the original FHS cohort, but the participant reported T2D family history during a follow-up exam, we used those records to expand available family information. For participants who were genotyped, genetic variant dosages from the Haplotype Reference Consortium release 1.1 based imputations were used in all analyses. Imputation was performed on the Michigan Imputation Server using minimac3 and the Haplotype Reference Consortium reference panel release 1.1 April 2016 using genetic variants passing the following criteria: call-rate ≥ 97%, Hardy–Weinberg *P* ≥ 10^–6^, < 1000 Mendelian errors, and MAF ≥ 1%. Prior to imputation, phasing was performed using the duoHMM algorithm incorporated into SHAPEIT2 to account for parental genotypes. We excluded variants with imputation quality r^2^ less than 0.3.

#### Incorporation of family history using LT-FH

From the full set of FHS participants, we retrieved T2D status and T2D family history of 8362 genotyped individuals, excluding participants of non-European ancestry, defined using principal component (PC) analysis.PCs were first computed with HapMap samples. Mean and standard deviation for PC1 and PC2 were computed for White samples, including those in FHS and CEU (Utah residents with ancestry from northern and western Europe). Participants were labeled as non-European if the value for PC1 or PC2 was greater than 6 standard deviations from the mean value for White samples. Using the LT-FH software, we calculated the posterior mean genetic liability scores for each participant based on the participant’s T2D status as well as any available parents’ or siblings’ T2D status. Then, we used a linear mixed-effects model to evaluate the association between T2D liability phenotypic scores and each imputed variant, adjusting for participant’s last exam age, sex, and the first ten PCs. We additionally adjusted for smoking status (never/former/current smoker) in a sensitivity analysis (see Additional File 1 for more details and results). We accounted for familial relatedness using a kinship matrix based on the FHS participants’ family structures.

#### Incorporation of family history using Fam-meta

We first identified two separate samples, one with probands (*n* = 8362) who have both T2D disease status and genotypes available, while the other included relatives (*n* = 3780) who only have T2D status but no genotypes available. For the first sample, we used a logistic mixed-effects regression model to evaluate the association between proband’s T2D case–control status and individual genetic variants, adjusting for last exam age, sex, and the first two PCs that were significantly associated with T2D. We accounted for relatedness using a kinship matrix based on the FHS pedigree. The first regression analysis corresponds exactly to CC-GWAS. In the second sample, we first matched the relatives with their corresponding probands using Framingham family identifiers. If a relative was related to multiple probands, the probands’ genotypes and phenotypes were averaged within the same family. Then, we used a logistic mixed-effects model to evaluate the association between relative’s T2D case–control status and imputed genetic variants based on probands’ genotypes, adjusting for proband’s phenotype, relative’s last exam age, relative’s sex, and the first two PCs. A kinship matrix was included to account for familial relatedness. For both regression models, we additionally adjusted for smoking status in a sensitivity analysis (see Additional File 1 for more details and results). Lastly, regression coefficients and variances from both regressions were combined using the meta-analysis formula provided in the original paper [6].

## Results

### Simulations

#### Results of type I error rate (all models)

**Fig. 1:**
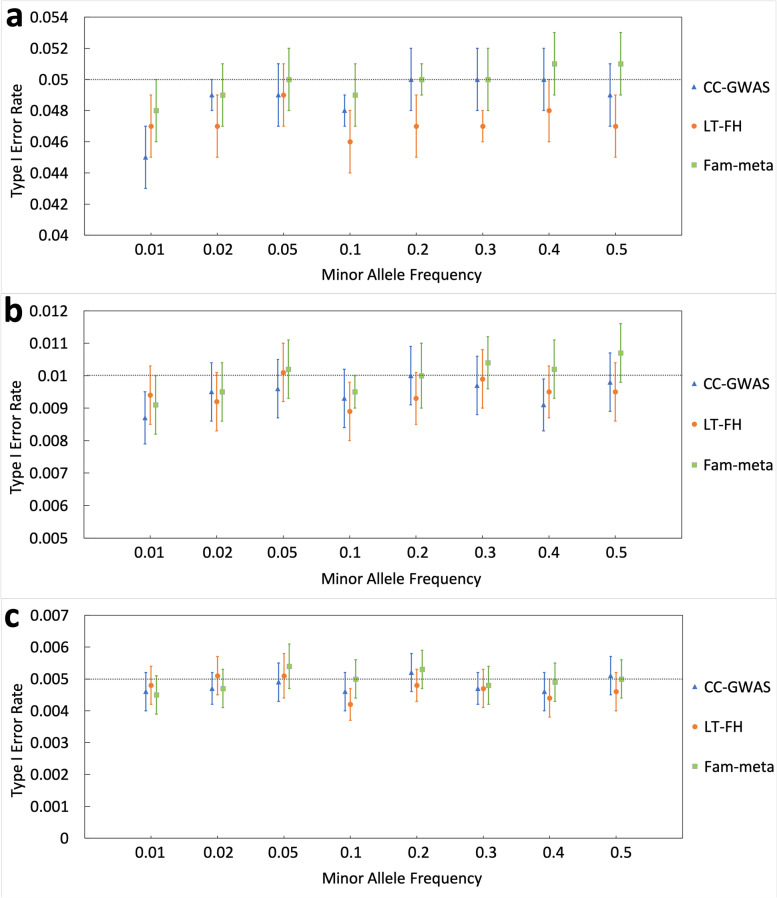
Type I error rate of CC-GWAS, LT-FH and Fam-meta using alpha levels of **a** 5%, **b** 1%, and **c** 0.5% using the following simulation parameters under model 1: The trait heritability was 0.28, and age and sex each explained 10% and 5% of the total variance, respectively. The sample size was 1800, consisting of 400 simple nuclear families. We ran 50,000 simulations under H_0_, and type I error rate was evaluated as proportion (%) of *P*-values less than alpha levels of 5%, 1% and 0.5% (indicated by dashed horizontal lines). Exact binomial 95% confidence interval was evaluated for each rate

Under model 1, we constructed a phenotype explained by age, sex, polygenic effects, and random error. Simulation results demonstrated a controlled type I error rate for all three approaches at alpha levels of 5%, 1% and 0.5%, i.e., the confidence intervals of error rates did not include the alpha levels (Fig. 1). In most simulated MAF scenarios, the type I error rates of LT-FH were lower than that of CC-GWAS and Fam-meta and than expected, illustrating that LT-FH was the most conservative method. For models 2 and 3 that included SNPs to generate the phenotype, the type I error rates were controlled as well (See Figures S2 and S3, Additional File 1).

#### Results of power (model 2)

In all simulated MAF scenarios, Fam-meta and LT-FH had higher power than CC-GWAS (Table 1), confirming our hypothesis that leveraging family information increases the power of genetic association analyses. In addition, Fam-meta was more powerful than LT-FH. Furthermore, simulations with less frequent causal variations yielded comparable increases in power from CC-GWAS to the two familial history methods (See Additional File 1 for more details and results). As genetic variant MAF increases, there was a greater increase in power from CC-GWAS to LT-FH and CC-GWAS to Fam-meta.

**Table 1:** Power of CC-GWAS, LT-FH and Fam-meta using the simulation parameters of model 2: we used a proportion of phenotypic variance explained by SNPs at 1% each; trait heritability was 0.28, while age and sex each explained 10% and 5% of the total variance, respectively. The sample size was 1800, consisting of 400 simple nuclear families. We ran a total of 5000 simulations under H_1_

Causal SNP minor allele frequency	CC-GWAS	LT-FH		Fam-meta	
		Power	Increase from CC-GWAS	Power	Increase from CC-GWAS
0.01	0.742	0.770	0.028	0.786	0.044
0.02	0.718	0.757	0.039	0.774	0.056
0.05	0.696	0.750	0.054	0.767	0.071
0.10	0.667	0.732	0.065	0.750	0.083
0.20	0.656	0.716	0.060	0.737	0.081
0.30	0.658	0.714	0.056	0.739	0.081
0.40	0.639	0.707	0.068	0.729	0.090
0.50	0.620	0.689	0.069	0.707	0.087

#### Results of power (model 3)

Using a phenotype model that included an interaction term between age and genotype of causal variants in addition to the causal variants, Fam-meta and LT-FH both remained more powerful than CC-GWAS. For causal variants with allele frequencies equal to and greater than 0.05, the increase in power from CC-GWAS to LT-FH and Fam-meta exceeded 10% (Table 2). Overall, the increase in power from CC-GWAS to the two familial methods was twice the increase when no interaction term was involved (Tables 1 and 2). This was expected because under the interaction model, the prevalence of disease increased with age. Therefore, for an offspring carrying the disease risk allele, disease phenotypes may not be expressed until at a much older age. As the offspring must have inherited the risk allele from one of his parents, the parent was more likely to be affected with the disease given his older age. However, the parent was less likely to have genotypes available, as they might be deceased due to aging or the disease. Under this scenario, leveraging the parents’ disease status provided greater information in association analyses with offspring, because the heritable disease would be detected more frequently in older family members.

**Table 2:** Power of CC-GWAS, LT-FH and Fam-meta under model 3 scenario with an interaction between age and genotype of causal variants. We ran a total of 5000 simulations

Causal SNP minor allele frequency	CC-GWAS	LT-FH		Fam-meta	
		Power	Increase from CC-GWAS	Power	Increase from CC-GWAS
0.01	0.789	0.841	0.052	0.861	0.072
0.02	0.750	0.832	0.082	0.864	0.114
0.05	0.732	0.838	0.106	0.859	0.127
0.10	0.718	0.833	0.115	0.854	0.136
0.20	0.695	0.822	0.127	0.850	0.155
0.30	0.686	0.814	0.128	0.846	0.160
0.40	0.684	0.819	0.135	0.842	0.158
0.50	0.660	0.806	0.146	0.831	0.171

#### Use of more distant relatives

While LT-FH could not incorporate grandparents’ disease status, Fam-meta allowed for more distant relatives, so we compared the power of Fam-meta using either two or four grandparents, along with the third generation offspring. Compared to the association power using parents’ disease status (Table 1), the overall power for Fam-meta decreased when using grandparents’ disease status (Table 3). For each simulated genetic variant, the power increased around 1–2% from CC-GWAS to Fam-meta when two grandparents were used. The increase in power from CC-GWAS to Fam-meta when using four grandparents (range between 0.02 to 0.05) was approximately half of the increase in power when using parents (range between 0.04 to 0.09) (Tables 1 and 3) but was approximately twice of the increase in power when using only two grandparents. Overall, this scenario demonstrated that incorporating the disease history of more distant relatives could still contribute to an increase in association power.

**Table 3:** Power of CC-GWAS and Fam-meta evaluated using more distant relatives’ information. Grandparents’ phenotypic status was used instead of parents’ phenotypic status for Fam-meta. We compared the power of Fam-meta when including two grandparents vs. four grandparents. LT-FH could not leverage second-degree relatives and thus results are not presented in this table

Causal SNP minor allele frequency	Fam-meta		Increase in power from CC-GWAS to Fam-meta	
	Two grandparents	Four grandparents	Two grandparents	Four grandparents
0.01	0.689	0.699	0.011	0.021
0.02	0.672	0.680	0.013	0.020
0.05	0.629	0.655	0.015	0.041
0.10	0.610	0.633	0.022	0.045
0.20	0.601	0.625	0.025	0.049
0.30	0.588	0.609	0.032	0.053
0.40	0.571	0.596	0.021	0.045
0.50	0.562	0.590	0.021	0.050

### Application to T2D using the FHS dataset

**Fig. 2:**
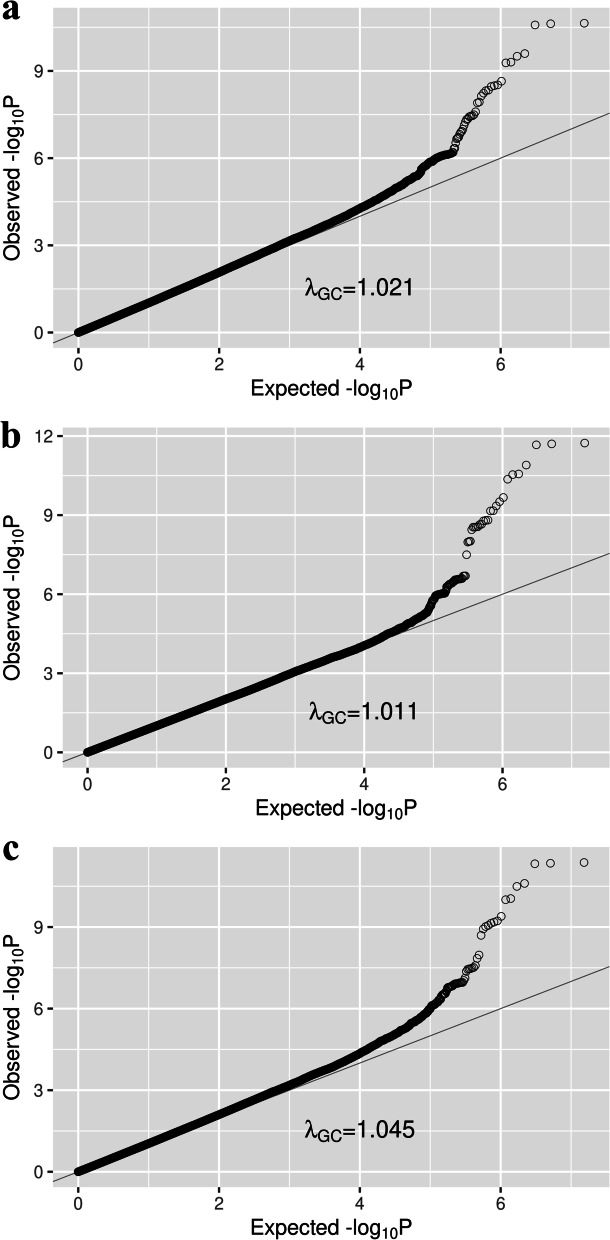
Quantile-Quantile plots of T2D association analysis results based on FHS data using **a** CC-GWAS, **b** LT-FH, and **c** Fam-meta. Each plot represents expected -log10 *P*-values vs. observed -log10 *P*-values. The gray line denotes y = x, and deviation from the line represents deviation of actual *P*-value from expected *P*-value under the null hypothesis. The genomic control factor λ_GC_ is also shown on each plot

**Fig. 3:**
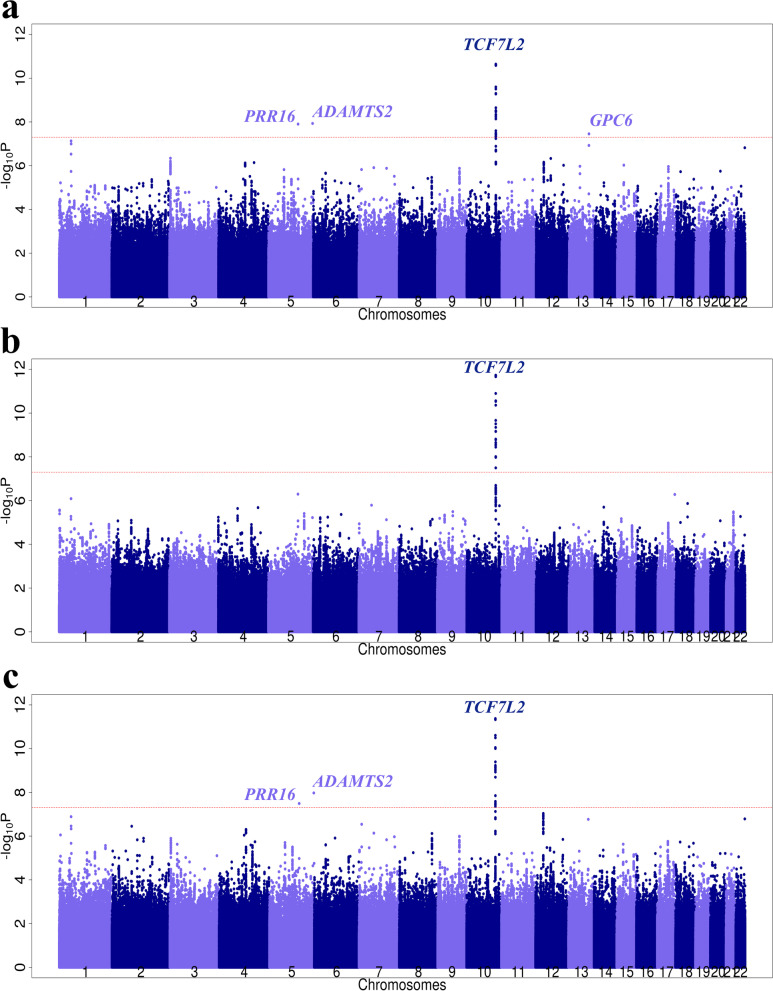
Manhattan Plots of T2D association analysis based on FHS data results using **a** CC-GWAS, **b** LT-FH, and **c** Fam-meta. For each method, the x-axis represents chromosome positions of tested variants across the genome, while the y-axis represents -log10 *P*-values for the association of genetic variants with T2D. The horizontal dashed red line in each figure represents the threshold for genome-wide significance, which corresponds to *P* = 5 × 10^–8^. All variants that passed the threshold are labelled with their corresponding gene names or closest reference gene names

A total of 7,006,827 variants passed our filters (excluding variants with imputation quality r^2^ less than 0.3 and MAF less than 0.01) and were included in the final analyses. Quantile–Quantile plots and Manhattan Plots of T2D association results using each familial history method, compared to CC-GWAS, are shown in Figs. 2 and 3. According to the genomic control inflation factors λ_GC_, there was little to no evidence of type I error inflation (λ_GC_ = 1.021 for CC-GWAS, λ_GC_ = 1.011 for LT-FH, and λ_GC_ = 1.045 for Fam-meta, see Fig. 2). Applying the genome-wide threshold of *P* < 5 × 10^–8^, we confirmed the strong association of the *TCF7L2* region with T2D using all three methods (Fig. 3). In addition, leveraging familial information increased the significance of the association at this locus compared to CC-GWAS. The top variant was rs7903146 (*P* = 2.0 × 10^–12^ for LT-FH and *P* = 4.5 × 10^–12^ for Fam-meta, compared to *P* = 2.3 × 10^–11^ for CC-GWAS, see Table 4).

**Table 4:** Main T2D associations from application to FHS data ordered by CC-GWAS *P*-value. The first four genetic loci have *P*-values less than the genome-wide significant threshold of *P* = 5 × 10^–8^ with at least one method, and the other two loci have been previously reported in literature to be associated with T2D and have *P*-values less than a suggestive threshold of *P* = 10^–3^ with at least one method

			CC-GWAS		LT-FH		Fam-meta	
rsIDs (Chr:b37Pos Mb)	Effect Allele and Frequency	Closest Gene Label	t-statistic	*P*-value	t-statistic	*P*-value	Tmeta	*P*-value
rs7903146 (10:114.8)	T (0.31)	*TCF7L2*	6.69	2.3 × 10^–11^	7.04	2.0 × 10^–12^	6.92	4.5 × 10^–12^
rs78825768 (5:178.7)	C (0.048)	*ADAMTS2*	5.70	1.2 × 10^–8^	4.53	6.0 × 10^–6^	5.72	1.1 × 10^–8^
rs150003225 (5:119.7)	C (0.012)	*PRR16*	5.69	1.3 × 10^–8^	5.02	5.1 × 10^–7^	5.53	3.2 × 10^–8^
rs78855997 (13:94.2)	G (0.010)	*GPC6*	5.51	3.5 × 10^–8^	4.20	2.6 × 10^–5^	5.23	1.7 × 10^–7^
rs2383208 (9:22.1)	G (0.18)	*CDKN2B-AS1*	-4.15	3.4 × 10^–5^	-4.50	6.8 × 10^–6^	-4.18	2.9 × 10^–5^
rs10830963 (11:92.7)	G (0.27)	*MTNR1B*	3.84	1.2 × 10^–4^	2.53	1.1 × 10^–2^	3.68	2.4 × 10^–4^

We also compared test statistics for all three methods for known T2D gene loci from published linkage and association studies. We extracted those loci and their corresponding *P*-values from our summary statistics. After filtering the *P*-values based on *P* < 10^–3^ for at least one method, three genetic regions remained: *TCF7L2*, *CDKN2B-AS1,* and *MTNR1B* (Table 4). No specific pattern was observed for the difference in significance between the three methods at either *CDKN2B-AS1* or *MTNR1B*.

**Fig. 4:**
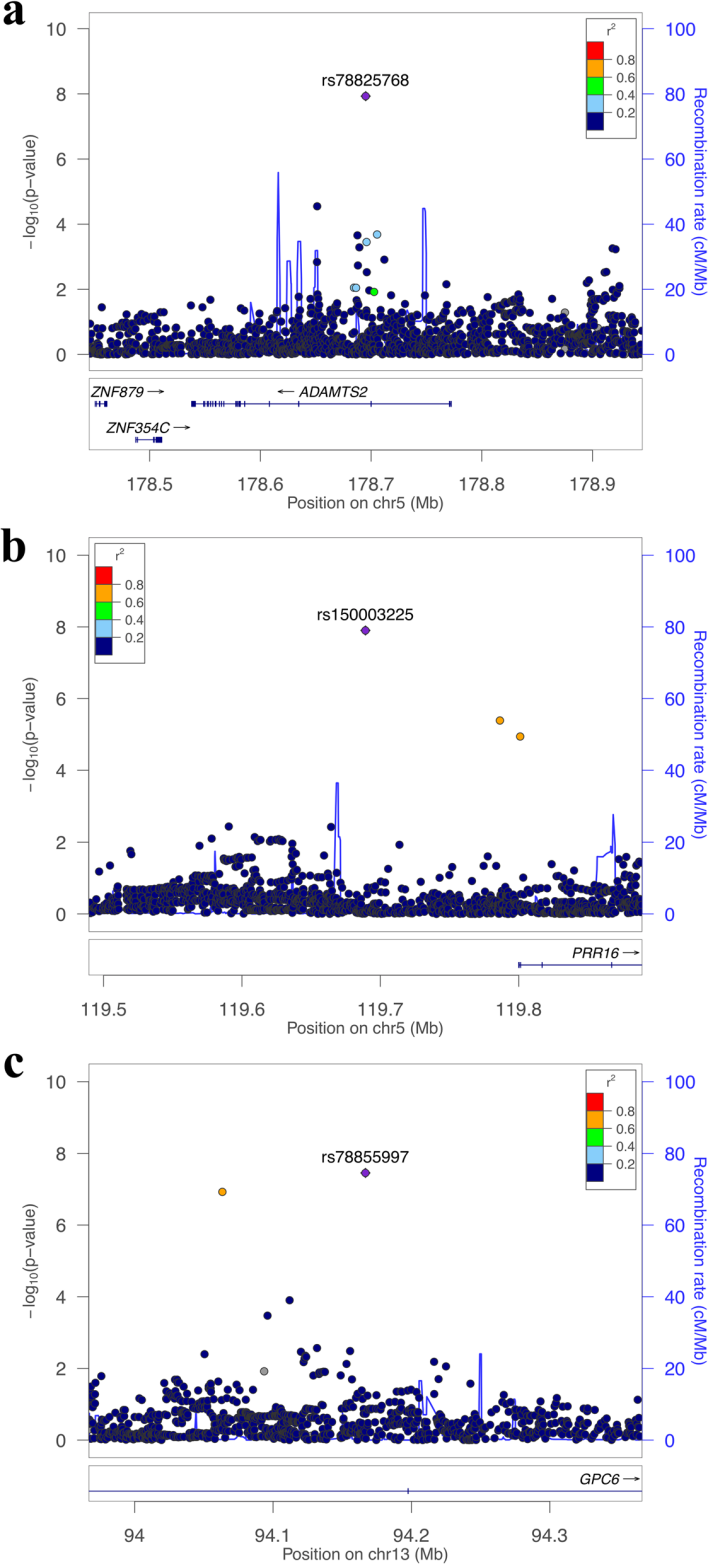
Regional association plots of novel loci **a** 5q35 (rs78825768) **b** 5q23 (rs150003225) and **c** 13q31 (rs78855997) using CC-GWAS results. LD was computed using the 1000 Genomes reference panel in the European population

**Fig. 5:**
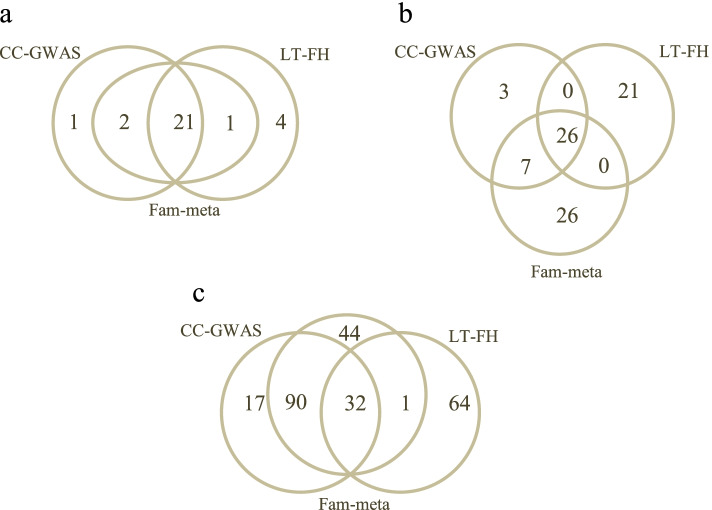
Venn diagram displaying the number of genetic variants that passed specific significant thresholds in CC-GWAS, LT-FH and Fam-meta. This figure presents the variant counts with *P*-values less than a specific threshold of **a** *P* = 5 × 10^–8^, **b** 5 × 10^–7^ and **c** 5 × 10^–6^ for each method, as well as the number of variants in common between each method

As for the other genetic variants that either passed or were close to the genome-wide significance threshold, we observed different patterns for each method. LT-FH did not detect any other regions at the genome-wide level besides the *TCF7L2* region; compared to CC-GWAS, there was a general decrease in variant significance across the genome (Fig. 3b). This trend may be explained by the conservativeness of LT-FH, as observed in our type I error simulations. In contrast, the Fam-meta association results were very comparable to that of the CC-GWAS (Fig. 3c). Using CC-GWAS and Fam-meta, we detected the same two novel low frequency variants at 5q35 (top association: rs78825768) and 5q23 (top association: rs150003225) (Fig. 4) that passed the genome-wide significance threshold and have not been previously reported in the literature to be associated with T2D. In addition, CC-GWAS was the only method that detected a low frequency variant (rs78855997) at the *GPC6* locus (Fig. 4), also not previously reported to be a T2D locus. To visualize the overlapping of results between the three methods, we compared the total number of variants passing the thresholds of 5 × 10^–8^, 5 × 10^–7^, and 5 × 10^–6^ for each method (Fig. 5). With all three thresholds, CC-GWAS and Fam-meta shared a higher number of variants compared to CC-GWAS and LT-FH (Fig. 5). In general, adding family history information resulted in a more drastic change in association results from CC-GWAS to LT-FH, given the overall reduction in the number of significant and near-significant genetic variants detected by LT-FH. The reduction in the number of significant variants was not observed when using Fam-meta. Lastly, our main results and conclusions did not change when adjusting for smoking status (see Additional File 1 for results of the sensitivity analysis).

## Discussion

We have examined two approaches, LT-FH and Fam-meta, that incorporate family history into genetic association studies of binary traits. As each approach was shown to be more powerful than CC-GWAS in their respective study, we compared them against each other using simulated data, and evaluated their performances in the association analysis of T2D in FHS.

From our simulations comparing LT-FH and Fam-meta to CC-GWAS, we concluded that Fam-meta was the most powerful, while LT-FH was the most conservative. Moreover, we observed a greater increase in power from CC-GWAS to the two familial methods when an interaction between age and genotype was added to the phenotype model. This indicates that LT-FH and Fam-meta are more powerful when age has a larger influence on the phenotype. In the scenario where we considered more distant relatives, the power increase from CC-GWAS to Fam-meta when using four grandparents’ phenotypic information was approximately twice of the increase in power when only two grandparents were used, but half of the increase in power when two parents were used. Our conclusions should still hold if SNPs in LD are analyzed, since both LT-FH and Fam-meta described in this paper evaluate the association of each variant individually, regardless of whether there is any correlation among the variants. Using data from the FHS, we detected several genome-wide significant hits. First, the *TCF7L2* region was detected by all three methods. Given *TCF7L2* is a well-known transcription factor that is consistently associated with T2D in Europeans and other ancestral groups [8], we expected to observe a strong association in our cohort as well. There was also a decrease in the *P*-value of the *TCF7L2* locus using each familial history method compared to using CC-GWAS, demonstrating that taking into account the disease status of relatives increased the power of detecting known genetic variants. Two loci at 5q35 and 5q23 (Fig. 4) that passed the genome-wide significant threshold with CC-GWAS and Fam-meta have not yet been reported as associated with T2D by previous studies, but appear to be particularly promising: promoter and enhancer histone marks are described in the adipose tissue [9] for the top association at 5q35 that lies in an intron of *ADAMTS2* (ADAM metallopeptidase with thrombospondin type 1 motif 2). *ADAMTS2* is primarily expressed in fibroblast cells, with low tissue specificity [10]. Associations have been detected between genetic variants within *ADAMTS2* and body fat distribution to the trunk [11], and downregulated *ADAMTS2* expression in heart tissues has been described in cardiac hypertrophy induced by pressure overload [12]. The 5q23 signal lies at 110 kb from the closest reference gene *PRR16* (proline rich 16), which encodes a cell-size regulator and is predominantly expressed in fibroblast cells [10]. Differential methylation levels at CpG sites within *PRR1*6 have been reported to be associated with maternal BMI and coronary heart disease [13]. Interestingly, the two loci were not detected by LT-FH at the same level of significance, and we believe this is due to the conservativeness of LT-FH. Lastly, the locus at 13q31 was detected by only CC-GWAS at genome-wide significance. The gene in which the top intronic variant resides, *GPC6* (glypican 6)*,* has higher levels of expression in the aortic and pituitary gland tissues [10]. Multiple associations in this gene have been reported for traits related to T2D, including triglycerides [14], pulse pressure [15], and smoking-T2D interaction [16].

Further examining known T2D loci previously identified in the literature, we did not observe any notable increase in significance using LT-FH and Fam-meta compared to CC-GWAS, besides the apparent *TCF7L2* region. However, considering that most study cohorts already collect family health history information, and LT-FH and Fam-meta are both easy to implement, it is only beneficial to include family history in association studies, even if the increase in power is small. We expect the gain in power to be much greater when analyzing a disease that is more strongly correlated with aging, such as T2D or dementia. If a study sample does not include many elderly participants, parents’ disease status can be much more informative for studying age-dependent penetrant diseases. Such a scenario was demonstrated by our interaction model in the simulations. In addition, LT-FH has been extended to LT-FH ++ that adopts an age-dependent liability threshold model, which accounts for disease age of onset in addition to family history [17].

In terms of practicality and application, LT-FH and Fam-meta both had their advantages as well as drawbacks. LT-FH provides a posterior genetic liability score even for individuals who had missing disease status, as long as the disease status was available for parents and/or siblings. In addition, the format required of sibling status was either “at least one sibling with disease” or “no disease”, so incomplete sibling status could still be informative in LT-FH. On the other hand, for Fam-meta, the logistic regressions required phenotypes available for all probands and relatives. Fam-meta could be modified to incorporate all relatives, while LT-FH could only use parents and siblings. Fam-meta was also easier to implement, because it is an extension of the logistic regression model (CC-GWAS). The LT-FH software was more computationally intensive when the sample size was large.

There are several limitations to our study. First, there could be some inaccuracy in the T2D family history information in the FHS that was self-reported. Secondly, when performing simulations, we needed to limit the sample size for each iteration because LT-FH was computationally intensive. Moreover, given the predominantly European ancestry of participants in the FHS cohort, our results may not generalize to other population groups. Finally, we did not study the effect of incorporating family history into rare variant association analysis. A recently published new method, family history aggregation unit-based test (FHAT), further extended LT-FH to analyze rare variants [18]. FHAT was able to detect genes with suggestive associations with several diseases in FHS [19]. Some additional work may include more extensive simulation scenarios, such as simulating more complex family structures, and adding more causal variants. One could also extend the application of both approaches to other diseases with different heritability, such as hypertension and lung cancer.

## Conclusions

In this paper, we examined two innovative approaches that extend GWAS to incorporate familial history, regardless of genotyping status. LT-FH uses liability threshold modeling, while Fam-meta meta-analyzes summary statistics from two logistic regressions. As presented throughout our analyses in simulations and application to FHS data, both LT-FH and Fam-meta outperformed, or performed equally well as CC-GWAS. Therefore, we highly recommend the inclusion of family disease history in GWAS using the two approaches addressed in this study. Researchers can use our results to determine whether LT-FH or Fam-meta is more suitable for their studies.

## Supplementary Information

**Additional file 1.** Additional Analyses. Description of data: Results of additional simulations and real-data analyses.

## Data Availability

This study uses data from the Framingham Heart Study, which are available in dbGaP via https://www.ncbi.nlm.nih.gov/projects/gap/cgi-bin/study.cgi?study_id=phs000007.v32.p13 (accession number: phs000007.v32.p13).

## Abbreviations

CC-GWAS: Logistic regression for assessing association with case–control status in genome-wide association studies
FHS: Framingham Heart Study
LT-FH: Liability threshold model conditional on both case–control status and family history
Fam-meta: Meta-analysis approach for leveraging family history in association studies
T2D: Type 2 diabetes
MAF: Minor allele frequency
LD: Linkage disequilibrium
PC: Principal component
FHAT: Family history aggregation unit-based test
