# Supplementary material for: Leveraging family history in genetic association analyses of binary traits
Source: BMC Genomics. 2022 Oct 1;23:678. doi: 10.1186/s12864-022-08897-8 (PMC9526325; doi:10.1186/s12864-022-08897-8)
Supplement: Supplementary file 1 — Additional file 1. Additional Analyses. Description of data: Results of additional simulations and real-data analyses. [file 12864_2022_8897_MOESM1_ESM.docx]

**Leveraging Family History in Genetic Association Analyses of Binary Traits**

**Additional File 1**

Yixin Zhang^1^, James B. Meigs^2,3^, Ching-Ti Liu^1^, Josée Dupuis^1,4^, Chloé Sarnowski^5^

^1^Department of Biostatistics, Boston University School of Public Health, Boston, Massachusetts, United States of America

^2^Division of General Internal Medicine, Massachusetts General Hospital, Boston, Massachusetts, United States of America

^3^Department of Medicine, Harvard Medical School, Boston, Massachusetts, United States of America

^4^Department of Epidemiology, Biostatistics and Occupational Health, McGill University, Montréal, Québec, Canada

^5^Department of Epidemiology, Human Genetics, and Environmental Sciences, The University of Texas Health Science Center, School of Public Health, Houston, Texas, United States of America

Correspondence to Yixin Zhang: [zhangyix@bu.edu](mailto:zhangyix@bu.edu)

Table of Contents

[1) Incorporating causal variants with smaller MAFs 2](#_Toc109991141)

[2) Realistic LD patterns simulations 3](#_Toc109991142)

[3) Additional details for simulating phenotype with a larger age effect 5](#_Toc109991143)

[4) False positive rate assessments for simulation models 2 and 3 6](#_Toc109991144)

[5) Sensitivity analysis examining the effect of smoking on real data association analysis results 7](#_Toc109991145)

1. ***Incorporating causal variants with smaller MAFs***

To investigate the influence of causal SNPs with less common alleles, we generated another group of eight causal variants with the following minor allele frequencies: 0.5%, 0.7%, 0.9%, 1%, 2%, 5%, 10%, and 15%. The minor allele counts of each variant were still equal to or more than 10. Then, we generated phenotypes using these eight variants under model 2 conditions (as described in the main paper), and determined the association between phenotypes and genotypes using CC-GWAS, LT-FH, and Fam-meta. Power for each method is shown in Table S1. The increase in power of the two familial history methods over CC-GWAS was comparable to what was observed with more frequent causal variants (MAFs between 1% and 50%) (Table 1 and Table S1).

| Causal SNP minor  allele frequency | CC-GWAS | LT-FH | | Fam-meta | |
| --- | --- | --- | --- | --- | --- |
|  |  | Power | Increase from CC-GWAS | Power | Increase from CC-GWAS |
| 0.005 | 0.742 | 0.763 | 0.021 | 0.775 | 0.033 |
| 0.007 | 0.735 | 0.766 | 0.031 | 0.780 | 0.045 |
| 0.009 | 0.736 | 0.765 | 0.029 | 0.779 | 0.043 |
| 0.01 | 0.731 | 0.756 | 0.025 | 0.773 | 0.042 |
| 0.02 | 0.715 | 0.761 | 0.046 | 0.777 | 0.062 |
| 0.05 | 0.684 | 0.727 | 0.043 | 0.751 | 0.067 |
| 0.1 | 0.681 | 0.741 | 0.060 | 0.758 | 0.077 |
| 0.15 | 0.649 | 0.710 | 0.061 | 0.727 | 0.078 |

Table S1. Power of CC-GWAS, LT-FH and Fam-meta using the simulation parameters of model 2 and less frequent causal SNPs. The MAF of each SNP was 0.5%, 0.7%, 0.9%, 1%, 2%, 5%, 10%, and 15%.

1. ***Realistic LD patterns simulations***

In this scenario, we incorporated genotypes that reflected realistic LD patterns in the genome. We first used the software HAPGEN2 and the 1000 Genome Phase 3 reference panel (EUR) to generate genetic data in a region between 16Mb to 32Mb from chromosome 21 for 5000 haplotypes, equivalent to genotypes for 2500 individuals. Then, 3600 haplotypes were randomly selected and assigned to 1800 families, with the same family structure as described for previous simulations. We randomly selected eight causal variants with MAFs distributed similarly to the previous group of independent variants: rs34579258 (MAF=0.01), rs112165276 (MAF=0.05), rs148408345 (MAF=0.11), rs4818385 (MAF=0.23), rs2824515 (MAF=0.24), rs55653623 (MAF=0.32), rs11701608 (MAF=0.42), and rs62215880 (MAF=0.50). Among these, rs2824515 and rs11701608 were in high LD with r^2^=0.98. We generated phenotypes using model 2 parameters (see main paper) and calculated power as described for model 2. In terms of type I error rate, we also used model 2 to generate phenotypes, but reduced the total phenotypic variance explained by variants to 0 and increased the proportion of variance explained by polygenic component to 28%. Then, we assessed the association between phenotypes and genotypes using all three methods and conducted 5000 iterations.

As in previous simulations, Fam-meta had the greatest power for all causal variants, while LT-FH was more powerful than CC-GWAS but less powerful than Fam-meta (Table S2). Since two highly correlated variants, rs2824515 and rs11701608, were both included in the generation of phenotypes, we expected an increase in their effective effect sizes i.e., in the phenotype model (see model 2), $\beta_{rs2824515}\left( g_{rs2824515} \right)+ \beta_{rs11701608}\left( g_{rs11701608} \right)$ was approximately equivalent to 2$\beta_{rs2824515}\left( g_{rs2824515} \right)$ or 2$\beta_{rs11701608}\left( g_{rs11701608} \right)$. For both variants, the power was greater than 90% for all three methods, while the power to detect an association for the other variants was similar to the power obtained when causal variants were independent as used previously for model 2 (Tables 1 and S2). Type I error rate was controlled for all three methods at an alpha level of 5% (Figure S1).

| Causal SNP  ID (Minor allele frequency) | CC-GWAS | LT-FH | | Fam-meta | |
| --- | --- | --- | --- | --- | --- |
|  |  | Power | Increase from CC-GWAS | Power | Increase from CC-GWAS |
| rs115684504 (0.013) | 0.778 | 0.813 | 0.035 | 0.833 | 0.056 |
| rs79866369 (0.051) | 0.641 | 0.680 | 0.039 | 0.704 | 0.063 |
| rs56268910 (0.110) | 0.686 | 0.735 | 0.050 | 0.754 | 0.068 |
| rs2824515 (0.235) | 0.956 | 0.984 | 0.028 | 0.990 | 0.034 |
| rs11701608 (0.242) | 0.956 | 0.984 | 0.028 | 0.989 | 0.032 |
| rs9306018 (0.322) | 0.653 | 0.713 | 0.060 | 0.735 | 0.082 |
| rs229062 (0.428) | 0.664 | 0.736 | 0.072 | 0.752 | 0.088 |
| rs8130153 (0.500) | 0.685 | 0.751 | 0.065 | 0.777 | 0.092 |

Table S2. Power of CC-GWAS, LT-FH and Fam-meta under model 2 scenario with some causal variants in LD.

Figure S1. Type I error rate at an alpha level of 5% (indicated by the dashed horizontal line) of CC-GWAS, LT-FH and Fam-meta under model 2 scenario with SNP effects at 0%. Exact binomial 95% confidence interval was evaluated for each rate.

1. ***Additional details for simulating phenotype with a larger age effect***

To simulate a phenotype that resembles late-onset diseases, we used model 2’s formula (shown in the main text), increasing the proportion of variance explained by age from 10% to 30%, while reducing the variance explained by the polygenic component from 57% to 37%. At an age effect of 10%, the difference in disease prevalence between parents and offspring was approximately 20%, while the difference increased to 35% when the age effect explained 30% of the variance in the disease liability. With an increased age effect, the power for all three approaches increased as well (Table S3). However, the increase in power from CC-GWAS to LT-FH and Fam-meta barely changed compared to when age effect was at 10%. Since changing the variance explained by age did not produce any noteworthy results, we additionally added an interaction term between age and genotype into the model. Results of the interaction model are shown in the main text.

| Causal SNP minor  allele frequency | CC-GWAS | LT-FH | | Fam-meta | |
| --- | --- | --- | --- | --- | --- |
|  |  | Power | Increase from CC-GWAS | Power | Increase from CC-GWAS |
| 0.01 | 0.802 | 0.811 | 0.009 | 0.840 | 0.038 |
| 0.02 | 0.769 | 0.786 | 0.017 | 0.825 | 0.056 |
| 0.05 | 0.733 | 0.764 | 0.031 | 0.809 | 0.076 |
| 0.10 | 0.704 | 0.748 | 0.044 | 0.804 | 0.100 |
| 0.20 | 0.667 | 0.722 | 0.055 | 0.777 | 0.110 |
| 0.30 | 0.658 | 0.722 | 0.064 | 0.772 | 0.114 |
| 0.40 | 0.643 | 0.709 | 0.066 | 0.764 | 0.121 |
| 0.50 | 0.620 | 0.688 | 0.068 | 0.746 | 0.126 |

Table S3. Power of CC-GWAS, LT-FH and Fam-meta under model 2 scenario, after increasing the age effect from 10% to 30%.

1. ***False positive rate assessments for simulation models 2 and 3***

To ensure that the difference in phenotype distribution simulated under models 2 (second phenotype model with eight variants contributing to liability) and 3 (third phenotype model with age by genotype interaction included in addition to the eight variants) did not contribute to an elevated type-I error, we assessed the association between eight randomly generated SNPs and phenotypes generated under each model. Figures of false positive rates are shown below.

Figure S2. Type I error rate of CC-GWAS, LT-FH and Fam-meta using alpha level of 5% (indicated by the dashed horizontal line) and the simulation parameters under model 2. Exact binomial 95% confidence interval was evaluated for each rate.

Figure S3. Type I error rate of CC-GWAS, LT-FH and Fam-meta using alpha level of 5% (indicated by the dashed horizontal line) and the simulation parameters under model 3. Exact binomial 95% confidence interval was evaluated for each rate.

1. ***Sensitivity analysis examining the effect of smoking on real data association analysis results***

As cigarette smoking may be a potential confounder in our T2D association analysis, we conducted a sensitivity analysis by including smoking status as an additional covariate and re-ran association tests for variants shown in our main association table (Table 4). Smoking status was classified in three categories: current smoker, former smoker, and non-smoker. For both CC-GWAS and LT-FH, the effect estimates were very similar when including smoking status as a covariate in the model (Tables S4 and S5). As for Fam-meta, because a pooled beta estimate was not part of the method, we compared the values of meta-analyzed T statistics and P-values (Tmeta and Pmeta). Both values did not change drastically for any variant when analyses were adjusted for smoking status (Table S6).

|  | | | CC-GWAS (no smoking) | | CC-GWAS (smoking) | |
| --- | --- | --- | --- | --- | --- | --- |
| rsIDs (Chr:b37Pos  Mb) | Effect Allele and Frequency | Closest Gene Label | Beta | P-value | Beta | P-value |
| rs7903146  (10:114.8) | T (0.31) | *TCF7L2* | 0.34 | 2.3x10^-11^ | 0.35 | 1.4x10^-11^ |
| rs78825768 (5:178.7) | C (0.048) | *ADAMTS2* | 0.65 | 1.2x10^-8^ | 0.65 | 1.4x10^-8^ |
| rs150003225  (5:119.7) | C (0.012) | *PRR16* | 1.09 | 1.3x10^-8^ | 1.07 | 2.7x10^-8^ |
| rs78855997  (13:94.2) | G (0.010) | *GPC6* | 1.02 | 3.5x10^-8^ | 1.03 | 3.4x10^-8^ |
| rs2383208  (9:22.1) | G (0.18) | *CDKN2B-AS1* | -0.25 | 3.4x10^-5^ | -0.26 | 2.1x10^-5^ |
| rs10830963  (11:92.7) | G (0.27) | *MTNR1B* | 0.20 | 1.2x10^-4^ | 0.21 | 1.1x10^-4^ |

Table S4. Effects of adjusting for smoking status on main T2D association results using CC-GWAS. In our real data application, we evaluated the association between T2D and each genetic variant adjusting for participant’s last exam age, sex, and the first two PCs. Here, we re-ran the association tests for variants presented in Table 4, incorporating smoking status as an additional covariate. Beta coefficients and P-values were used for comparison between two models.

|  | | | LT-FH (no smoking) | | LT-FH (with smoking) | | |
| --- | --- | --- | --- | --- | --- | --- | --- |
| rsIDs (Chr:b37Pos  Mb) | Effect Allele and Frequency | Closest Gene Label | Beta | P-value | | Beta | P-value |
| rs7903146  (10:114.8) | T (0.31) | *TCF7L2* | 0.0513 | 2.0x10^-12^ | | 0.0518 | 1.2x10^-12^ |
| rs78825768 (5:178.7) | C (0.048) | *ADAMTS2* | 0.0826 | 6.0x10^-6^ | | 0.0817 | 7.5x10^-6^ |
| rs150003225  (5:119.7) | C (0.012) | *PRR16* | 0.175 | 5.1x10^-7^ | | 0.174 | 5.6x10^-7^ |
| rs78855997  (13:94.2) | G (0.010) | *GPC6* | 0.161 | 2.6x10^-5^ | | 0.161 | 2.6x10^-5^ |
| rs2383208  (9:22.1) | G (0.18) | *CDKN2B-AS1* | -0.0393 | 6.8x10^-6^ | | -0.0397 | 5.6x10^-6^ |
| rs10830963  (11:92.7) | G (0.27) | *MTNR1B* | 0.0201 | 1.1x10^-2^ | | 0.0200 | 1.2x10^-2^ |

Table S5. Effects of adjusting for smoking status on main T2D association results using LT-FH. In our real data application, we evaluated the association between T2D and each genetic variant adjusting for participant’s last exam age, sex, and the first ten PCs. Here, we re-ran the association tests for variants presented in Table 4, incorporating smoking status as an additional covariate. Beta coefficients and P-values were used for comparison between two models.

|  | | | Fam-meta (no smoking) | | Fam-meta (with smoking) | |
| --- | --- | --- | --- | --- | --- | --- |
| rsIDs (Chr:b37Pos  Mb) | Effect Allele and Frequency | Closest Gene Label | Tmeta | Pmeta | Tmeta | Pmeta |
| rs7903146  (10:114.8) | T (0.31) | *TCF7L2* | 6.92 | 4.5x10^-12^ | 6.95 | 3.7x10^-12^ |
| rs78825768 (5:178.7) | C (0.048) | *ADAMTS2* | 5.72 | 1.1x10^-8^ | 5.70 | 1.2x10^-8^ |
| rs150003225  (5:119.7) | C (0.012) | *PRR16* | 5.53 | 3.2x10^-8^ | 5.46 | 4.8x10^-8^ |
| rs78855997  (13:94.2) | G (0.010) | *GPC6* | 5.23 | 1.7x10^-7^ | 5.23 | 1.7x10^-7^ |
| rs2383208  (9:22.1) | G (0.18) | *CDKN2B-AS1* | -4.18 | 2.9x10^-5^ | -4.30 | 1.7x10^-5^ |
| rs10830963  (11:92.7) | G (0.27) | *MTNR1B* | 3.68 | 2.4x10^-4^ | 3.71 | 2.1x10^-4^ |

Table S6. Effects of adjusting for smoking status on main T2D association results using Fam-meta. In our real data application, we evaluated the association between T2D and each genetic variant adjusting for participant’s last exam age, sex, and the first two PCs. Here, we re-ran the association tests for variants presented in Table 4, incorporating smoking status as an additional covariate. Meta-analyzed T statistics and P-values (Tmeta and Pmeta) were used for comparison between two models.
